# Toward Nanodisc Tailoring for SANS Study of Membrane Proteins

**DOI:** 10.3390/bioengineering13010087

**Published:** 2026-01-12

**Authors:** Krishna Chaithanya Batchu, Mark D. Tully, Anne Martel

**Affiliations:** 1 Institut Max Von Laue and Paul Langevin, 38042 Grenoble, France; batchu@ill.fr; 2 European Synchrotron Radiation Facility, 38043 Grenoble, France; mark.tully@esrf.fr

**Keywords:** nanodiscs, small-angle scattering, membrane mimicks, contrast variation

## Abstract

Although membrane proteins are of major importance in both physiology and disease, they remain less studied than soluble proteins due to the complex amphiphilic environments required to preserve their structure and function. As a consequence, membrane proteins are under-represented in structural databases. In this work, we present a robust structural characterization of lipid nanodiscs designed to facilitate membrane protein studies by small-angle neutron scattering. By combining small-angle X-ray and neutron scattering, we investigate nanodiscs of three different sizes and three lipid compositions to accommodate a broad range of systems. Specifically, nanodiscs with diameters of approximately 9 nm, 12 nm, and 15 nm were examined. Beyond the commonly used di-myristoyl-phosphatidylcholine lipid, we produced and characterized polar lipid extracts from a Gram-negative bacterium (*Escherichia coli*) and a Gram-positive bacterium (*Bacillus subtilis*) under both protonated and deuterated conditions. In conclusion, solubility-enhanced variants of the scaffold protein yield more stable nanodiscs and are therefore preferable for extended structural investigations. The co-fitting of small-angle scattering data provides robust geometrical models of these nanodiscs, which can be treated as well-defined reference systems for future studies of membrane proteins in native-like lipid environments.

## 1. Introduction

Membrane proteins mediate communication between living cells and their environment. Owing to this central role, many are implicated in disease and constitute major therapeutic targets; consequently, beyond their fundamental biological importance, elucidating their structure, dynamics, and mechanisms can directly inform drug discovery. Despite this significance, membrane proteins remain more challenging to study than soluble proteins because they require a complex milieu—simultaneously hydrophobic and hydrophilic—to maintain their native structure and function. As a result, our understanding of membrane proteins remains limited, and the development of new methodological tools is continually required [1].

Over the past two decades, solution-based techniques such as nuclear magnetic resonance (NMR), cryo-electron microscopy (cryo-EM), and small-angle scattering (SAS) have benefited substantially from the use of nanodiscs: nanoscale lipid bilayers encircled by a scaffold protein or polymer that provide a near-physiological environment for membrane proteins [2,3,4]. Within nanodiscs, membrane protein structure and dynamics can differ markedly from those observed in detergent micelles, underscoring the critical influence of the lipid environment [5,6]. Nanodiscs are particularly valuable because they allow precise control over lipid composition while providing simultaneous access to both sides of the membrane.

Among solution techniques, small-angle neutron scattering (SANS) is especially well suited for probing macromolecular complexes and protein–lipid interactions [7,8,9]. This strength arises from isotopic contrast variation: protons and deuterons exhibit markedly different neutron scattering lengths while maintaining nearly identical chemical properties. Selective deuteration therefore enables the tuning of scattering length densities (SLDs), modulation of contrast, and isolation of specific component contributions within complex assemblies, with minimal perturbation to molecular structure or function [10,11]. As examples, contrast variation SANS has been used to study protein–protein complexes [12], protein–nucleic acid complexes [13], and protein–surfactant complexes [7,9,14].

Exploiting this principle, deuterated nanodiscs can be engineered to be contrast-matched in D_2_O-based buffers, thereby rendering them effectively invisible to neutrons. This approach allows protonated membrane proteins reconstituted within such nanodiscs to be studied in a near-native lipid environment using SANS signals that report predominantly on the protein itself. These so-called “stealth” nanodiscs were first developed and applied by Maric et al. to investigate an ABC transporter embedded in a POPC bilayer [15,16].

Building on this pioneering work, the present study aims to systematically characterize nanodiscs of varying sizes and lipid compositions to accommodate a broad range of membrane proteins. Nanodiscs were assembled using three scaffold (belt) proteins—MSP1D1, csE3, and spNW15—and three lipid formulations: pure dimyristoylphosphatidylcholine (DMPC), *Escherichia coli* polar lipid extract (EcLip), and *Bacillus subtilis* polar lipid extract (BsLip). This design yielded five nanodisc systems: MSP1D1–DMPC, spNW15–DMPC, csE3–DMPC, csE3–EcLip, and csE3–BsLip.

Each nanodisc composition was prepared in both protonated (h-) and deuterated (d-) forms, with the latter engineered to achieve contrast matching in D_2_O-based buffers. Protonated nanodiscs were structurally characterized using both SANS and small-angle X-ray scattering (SAXS), while the precise contrast-matching conditions of the deuterated nanodiscs were determined by SANS. Collectively, these results establish a robust experimental framework for future membrane protein studies in nanodiscs—including those employing native lipid extracts—and provide practical guidance for scaffold protein selection. 

## 2. Materials and Methods

### 2.1. Sample Preparation

#### 2.1.1. Reagents and DMPC

TBS buffer (50 mM Tris, 137 mM NaCl, pH 7.6; ref. 524750), D_2_O, n-dodecyl-β-D-maltoside (DDM; ref 850520P), methylated β-cyclodextrin (MeβCD; ref 332615), and 1,2-dimyristoyl-sn-glycero-3-phosphocholine (DMPC; ref 850345P) were purchased from Merck and used without further purification. Heavy water (D_2_O 99.9% purity) and all the HPLC-grade solvents including CHCl_3_ (99.5% purity), C_2_H_5_OH (98% purity), C_3_H_8_O (98% purity), and CH_3_OH (99.8% purity) were purchased from Sigma-Aldrich and used without any further purification. Glycerol-d8 was obtained from Eurisotop, France. Stationary phase silica columns for solid phase extraction and a semi-preparative Nucleosil 100-5 OH column, were purchased from Macherey-Nagel, Hœrdt, France. All other chemicals and cell culture media components were obtained (in the highest commercially available purity) from Sigma Aldrich, Saint-Quentin-Fallavier, France.

#### 2.1.2. Belt Proteins

MSP1D1 [2] is engineered to form nanodiscs with an approximate diameter of 90 Å. It carries an N-terminal His_7_ tag that can be cleaved by tobacco etch virus (TEV) protease and is not covalently circularized. The corresponding pET28a plasmid (kanamycin resistance, IPTG-inducible promoter) was obtained from Addgene (ref. #20061) and kindly provided by C. Julian Binard (ISBG, Grenoble, France).

csE3 is derived from MSP1E3D1, which forms nanodiscs with a nominal diameter of ~120 Å. This variant contains mutations that increase its net negative charge and solubility. Both His-tags are removed, and the protein is covalently circularized during purification through sequential enzymatic treatment with TEV protease and sortase. The pET28a plasmid was designed and provided by N. T. Johansen [17] (Addgene ref. #240152).

spNW15 and spNW30 (pET28a vectors; Addgene refs. #173483 and #173485) are expressed as covalently circularized scaffold proteins using the SpyCatcher system; their His-tags are retained. These constructs were designed as described in [18].

All scaffold proteins were overexpressed in *Escherichia coli* BL21(DE3) using M9++ minimal medium [19] supplemented with 10 g/L glycerol as the carbon source, 50 µg/mL kanamycin, and 1 mM. Fully protonated proteins (h-proteins in H_2_O-based media, whereas partially deuterated proteins (d-proteins) were expressed in media containing 85% D_2_O. No acclimation step was required for growth in 85% D_2_O, although growth rates were modestly reduced compared to H_2_O-based cultures. Induction and purification protocols followed previously reported procedures specific to each scaffold protein.

The final gel-filtration step was performed in 1 M ammonium bicarbonate buffer (pH 8.0). Purified proteins were aliquoted, lyophilized, and stored at −20 °C. Deuteration levels, determined by mass spectrometry (ISBG platform: https://www.isbg.fr/mass-spectrometry/ (accessed on 6 December 2025), ranged from 72% to 75%.

#### 2.1.3. Total Lipid Extraction

Polar lipid mixtures were extracted from *Escherichia coli* BL21(DE3) and *Bacillus subtilis* (strain 168) cultures grown at 37 °C with shaking at 180 rpm. Cells were harvested by centrifugation during the exponential growth phase and stored at −80 °C until use. Cultures were grown in either protonated or fully deuterated M9++ minimal medium, using H_2_O or D_2_O as the solvent and glycerol or d_8_-glycerol as the carbon source, respectively. Fully deuterated growth required a brief acclimation period following the protocol described by Cai et al. [19].

Frozen cell pellets were resuspended in 10 mL of Milli-Q H_2_O (18 MΩ·cm at 25 °C, Millipore) and lysed by probe sonication on an ice bath (3 × 5 min, 30 s intervals, 20% duty cycle). The resulting lysate was immediately poured into ethanol preheated to 65 °C containing 1% (*w*/*v*) butylated hydroxytoluene (BHT), followed by vigorous stirring to denature endogenous lipases.

Total lipids were subsequently extracted using the Bligh and Dyer method [20], as well as the protocol of Folch et al. [21]. The organic phase was evaporated under a stream of argon, and the dried lipid extract was finally redissolved in chloroform (CHCl_3_).

#### 2.1.4. Polar Mixture Fractionation by Preparative HPLC

Separation of the polar lipid mixtures was achieved through a two-step purification strategy. First, samples were fractionated using an amino-bonded solid-phase extraction (SPE) column. The eluate containing the targeted lipid classes was then further separated by high-performance liquid chromatography (HPLC) using a diol-modified silica stationary phase coupled to an Agilent 1260 Infinity II chromatographic system (Agilent Technologies, Les Ulis, France) equipped with a SEDEX 90 evaporative light-scattering detector (ELSD; Sedere, Alfortville, France).

Prior to injection, samples were dried under a stream of argon and redissolved in chloroform (CHCl_3_). Separation was performed on a semi-preparative Nucleosil 100–5 OH column (10 × 250 mm; Macherey-Nagel, Hœrdt, France). Elution was carried out at a flow rate of 1.0 mL/min using a binary solvent gradient composed of solvent A (CHCl_3_/CH_3_OH, 70:25 *v*/*v*, supplemented with 1% NH_4_OH) and solvent B (CHCl_3_/CH_3_OH/H_2_O, 60:40:5.5 *v*/*v*/*v*, supplemented with 0.5% NH_4_OH).

The gradient program was as follows: solvent B was held at 0% at injection, increased linearly to 40% over 30 min, then ramped to 100% at 40 min and maintained until 65 min. The gradient was subsequently returned to 0% solvent B at 66 min and held until the end of the run at 75 min. Throughout all measurements, the column was maintained at room temperature, and the ELSD drift tube temperature was set to 60 °C. Nitrogen was used as the carrier gas at an inlet pressure of 3.5 bar. Chromatographic data were processed using the OpenLab workstation software (Agilent Technologies, Les Ulis, France).

#### 2.1.5. Fatty Acid Methyl Esters (FAMEs) Analysis by GC-FID

The acyl chain composition of each purified lipid mixture was determined by gas chromatography with flame ionization detection (GC–FID) following hydrolysis of ester bonds and derivatization of the released fatty acids to their corresponding fatty acid methyl esters (FAMEs). Lipid extract films (0.1–1 mg) were derivatized by adding approximately 3 mL of methanolic HCl and incubating the mixture at 85 °C for 1 h.

GC analyses were performed using a GC-2010 Plus instrument (Shimadzu, Kyoto, Japan) equipped with a split/splitless injector and a BPX70 capillary column (70% cyanopropyl polysilphenylene-siloxane; 25 m × 0.22 mm inner diameter). Helium was used as the carrier gas at a flow rate of 1.04 mL/min, corresponding to a linear velocity of 35 cm/s, with a purge flow of 1 mL/min.

Prior to injection, the column was equilibrated at 155 °C for 3 min. The oven temperature was then increased to 180 °C at a rate of 2 °C/min, followed by a ramp to 220 °C at 4 °C/min, and held at 220 °C for 5 min, resulting in a total run time of 27.5 min. Samples (5 µL) were injected at 250 °C using an AOC-20i autosampler (Shimadzu).

Detection was performed using a flame ionization detector operated at 260 °C, with hydrogen, compressed air, and helium make-up gas flow rates of 40 mL/min, 400 mL/min, and 30 mL/min, respectively. Chromatographic data were processed using LabSolutions software (Shimadzu, Kyoto, Japan), which was used to identify and integrate chromatographic peaks and calculate the molar fraction of each FAME species.

#### 2.1.6. Nanodisc Assembly

The nanodisc assembly protocol was adapted from a previously established procedure [22]. Briefly, a total of 10 µmol of lipids were dissolved in 970 µL of TBS containing 20 µmol of n-dodecyl-β-D-maltoside (DDM) by performing five freeze–thaw cycles alternating between 8 °C and 45 °C. The lyophilized belt protein was then added to the lipid–DDM mixture at the following target molar ratios: 1 MSP1D1 per 75 lipids, 1 csE3 per 130 lipids, 1 spNW15 per 180 lipids, or 1 spNW30 per 250 lipids. The mixture was incubated for 45 min at 8 °C under magnetic stirring.

Subsequently, 30 µmol of methyl-β-cyclodextrin (MeβCD) was added to reach a final volume of 1 mL, and detergent removal—thereby triggering nanodisc assembly—was allowed to proceed overnight at 8 °C. Most conditions resulted in slightly turbid solutions; samples were therefore centrifuged for 15 min at 20,000× *g* and 10 °C prior to purification by size-exclusion chromatography (SEC) using a Superdex 200 Increase 10/300 column.

Note: Nanodisc assembly is generally more efficient above the lipid gel–fluid phase transition temperature (>24 °C for DMPC and >37 °C for *E. coli* and *B. subtilis* lipid extracts). However, low-temperature reconstitution was explored here to accommodate temperature-sensitive membrane proteins. Under these conditions, SEC revealed that spNW30–DMPC assemblies did not yield a homogeneous nanodisc population; consequently, this composition was not analyzed further. Proper assembly of such large nanodiscs may require temperatures above the lipid melting transition.

### 2.2. SANS Measurements

Small-angle neutron scattering (SANS) measurements were performed on the D22 instrument at the Institut Laue–Langevin (ILL, Grenoble, France) using standard symmetric configurations with sample-to-detector distances of 5.6 m or 8 m, complemented by a second detector positioned 1.4 m from the sample. A neutron wavelength of 6 Å (±10%) was used, with a 55 mm × 40 mm source aperture and a 10 mm × 7 mm sample aperture.

Samples were loaded into 1 mm pathlength Suprasil quartz cuvettes (Hellma, ref. 100-1-40) and measured at temperatures ranging from 14 to 24 °C, i.e., below the lipid melting temperature. Data reduction was performed using the GRASP software package, including corrections for sample thickness and neutron absorption, normalization to absolute intensity using direct beam flux measurements, and subtraction of empty-cell scattering and ambient background noise.

Subsequently, buffer scattering was subtracted using Igor Pro macros developed by S. Kline (NCNR, Gaithersburg, MD, USA). Data fitting was performed without concentration scaling; however, within each contrast series, the sample concentration was maintained constant.

### 2.3. SAXS Measurements

Small-angle X-ray scattering (SAXS) data were collected on the BM29 BioSAXS beamline at the European Synchrotron Radiation Facility (ESRF, Grenoble, France; https://www.esrf.fr/BM29-BioSAXS (accessed on 6 December 2025) [23]) using the standard automated sample changer and a 1 mm diameter quartz flow-through capillary. Data reduction was performed automatically using the DAHU pipeline [24].

### 2.4. SAS Data Analysis Based on Analytical Models

Small-angle scattering (SAS) curves of the nanodiscs were fitted using SASView version 6.0.0 (http://www.sasview.org/ (accessed on 6 December 2025)) with the “ellipsoid core–shell cylinder with rough belt” model. Parameter optimization was performed using the DREAM fitting algorithm with 100,000 samples, while all other settings were kept at their default values.

#### 2.4.1. SANS Data Fitting

SANS curves were fitted over a q-range of 0.01 to 0.25 Å^−1^. The scale factor was constrained to be identical across all contrasts for each nanodisc, maintaining constant concentration throughout. Geometrical parameters—including radius, ellipticity, length, rim thickness, and face thickness—were constrained within each contrast series. To minimize parameter correlation, the scattering length density (SLD) of the hydrophobic core of each disc was fixed to a single value across all contrasts, reflecting its lack of hydration. Buffer and protein SLDs were fixed to values calculated using the PSLDC calculator (http://psldc.isis.rl.ac.uk/Psldc/ (accessed on 6 December 2025)), assuming a 90% exchange of protein labile hydrogens with the solvent, and based on the following sequences:

**MSP1D1:**

GSTFSKLREQLGPVTQEFWDNLKEELRQEMSKDLEEKAKVQPYLDDFQKKWQEEMELYRQKVEPLRAELQEGARQKLHELQEKLSPLGEEMRDRARAHVDALRTHAPYSDELRQRLAARLEALKENGARLAEYHKATEHLSTLSEKAKPALEDLRQGLLPVLESFKVSFLSALEEYTKKLNTQ

**csE3:**

GSSFSKLREELGPVSEEFWDDLEKESEGLREEMSKDLEEVKAKVEPYLDDFEKKWEEEMELYREKVEPLRAELEEGAREKLHELEEKLSPLGEEMRDRARAHVDALRSHLAPYLDDFEKKWEEEMELYREKVEPLRAELEEGAREKLHELEEKLSPLGEEMRDRARAHDALRSHAPYSDELRERLAARLEALKEDGGARLAEYHAKASEHLSSLSEKAKPALEDLREGLLVLESFKVSFLSALEEYSKKLDSEGGRGGSLPET

**spNW15:**

GSSHHHHHHSSGLVPRGSHMASMTGGQQMGRGSGAMVTTLSGLSGEQGPSGDMTTEEDSATHIKFSKRDEDGRELAGATMELRDSSGKTISTWISDGHVKDFYLYPGKYTFVETAAPDGYEVATAITFTVNEQGQVTVNGEATKGDAHTSTFSKLREQLGPVTQEFWDNLEKETEGLRQEMSKDLEEVKAKVQPYLDDFQKKWQEEMELYRQKVEPLRAELEGARQKLHELQEKLSPLGEEMRDRARAHVDALRTHLAPYSDELRQRLAARLEALKENGGARLAEYHAKATEHLSTLSEKAKPALEDLRQGLLPVLESFKVSFLSALEEYTKKLNTQLPGTGAAALEVPTIVMVDAYKRYK

#### 2.4.2. SAXS Data Fitting

After re-estimating the error bars using the Bayesian indirect Fourier transform method [25], the three SAXS curves were simultaneously fitted using the same model and q-range as for the SANS curves. An exception was made for the spNW15–DMPC nanodiscs, where low-q data below 0.035 Å^−1^ were excluded due to evident effects of attractive interparticle interactions. Lipid-related parameters—including face thickness, hydrophobic core length, face SLD, and core SLD—were constrained to be identical across the three DMPC nanodiscs. The solvent SLD was fixed at 9.4 × 10^−6^ Å^−2^.

## 3. Results

### 3.1. Protonated DMPC Nanodiscs

**Figure 1:**
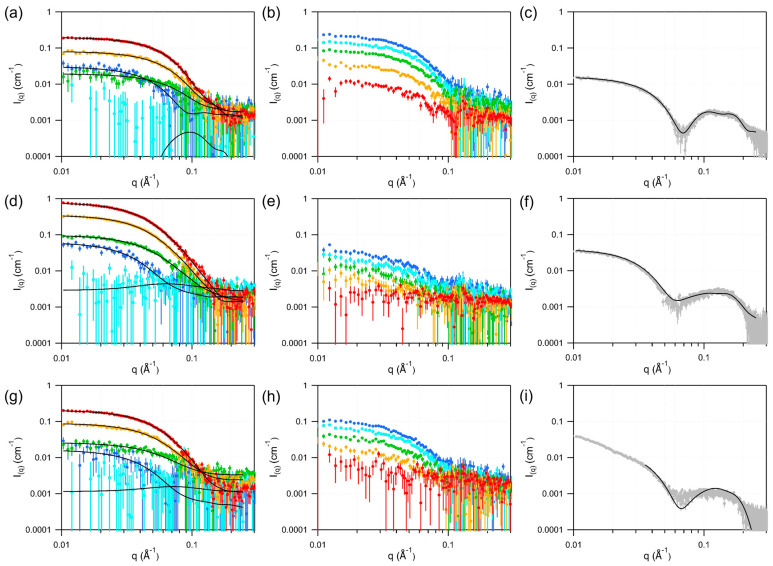
SAXS and SANS analysis of the DMPC nanodiscs. The first line (**a**–**c**) concerns nanodiscs surrounded by MSP1D1, the second line (**d**–**f**), by csE3, and the 3rd line (**g**–**i**) by spNW15. The first column shows the SANS curves of the protonated nanodiscs contrast series, the second column, the SANS curves of the deuterated nanodiscs contrast series, and the third column, the SAXS curves of the protonated nanodiscs in H_2_O-based buffer. On the first and second columns, the colors represent the buffer D_2_O content: red: 80%; yellow: 60%; green: 40%; light blue: 20%; dark blue: 0%. The continuous black lines are the fits to the data obtained using SASView and correspond to the parameters listed in Table 1.

Protonated DMPC nanodiscs were measured by SANS using five-point contrast variation and by SAXS in H_2_O-based buffer, with the resulting data presented in Figure 1a,d,g. To accurately determine the nanodisc geometry, all five SANS curves within each contrast series were simultaneously fitted under the constraint that the overall scale (proportional to concentration), hydrophobic core SLD, and all geometric parameters were identical across the series. Protein and solvent SLDs were fixed to calculated values using BSLDC (Myatt and Clifton, http://psldc.isis.rl.ac.uk/Psldc/ (accessed on 6 December 2025)), while the lipid headgroup and protein SLDs were allowed to vary to account for hydration effects and hydrogen exchange between the protein and solvent. SAXS profiles of the three nanodisc types were then fitted simultaneously, with constraints enforcing shared SLDs for the buffer, DMPC headgroups, and aliphatic chains across datasets (Figure 1c,f,i), ensuring consistent solvent and lipid contrasts in the modeling. Best-fit curves are shown as black lines, and the resulting parameter values—defined in a following figure—are summarized in Table 1 along with the normalized χ^2^ for each fit.

Independent analysis of SAXS and SANS data, each using tailored constraints, allowed cross-validation of the extracted structural parameters. Notably, the SAXS measurements were collected three days after the SANS experiments, with samples stored at room temperature, providing a qualitative assessment of relative stability across the three nanodisc types. Several differences and consistencies emerged from the two methods.

For hMSP1D1–hDMPC nanodiscs, SANS indicates a circular disc with a lipid-core radius of ~35 Å and a total diameter of 87 Å, slightly smaller than the 98 Å radius previously reported by SAXS [2]. These values are similar to those determined by Nakano et al. [26] by SANS, though the present analysis provides a more reliable phase distribution (hydrophobic core, lipid headgroups, and belt protein) due to multi-contrast fitting. The SAXS-derived parameters are broadly consistent with the SANS results.

For hcsE3–hDMPC nanodiscs, SAXS and SANS results agree closely, matching previously published values [17]: a radius of ~66 Å with strong ellipticity, a belt thickness slightly above 9 Å, a lipid headgroup layer of ~7.5 to 8 Å, and a hydrophobic core thickness of 31–32 Å. The close agreement between independent SAXS and SANS fits confirms that the MSP1E3D1 modifications—increased negative charge and covalent circularization—enhance disc stability.

In the case of hspNW15–hDMPC nanodiscs, signs of aging were apparent in the SAXS profiles, necessitating exclusion of low-q data. Within the usable range, SAXS and SANS fits yield consistent parameters, indicating a slightly smaller yet still elliptical disc compared to csE3–DMPC nanodiscs.

Parameters refined from SANS data fitting of DMPC discs show that the headgroup SLDs increase with the D_2_O solvent content, suggesting a propensity to hydrate. The aliphatic chains SLD is very close to the theoretical value [27,28]. SAXS data fitted values are also in line with previously published comparable data [29].

### 3.2. Protonated Natural Lipid Mixes Nanodiscs

**Figure 2:**
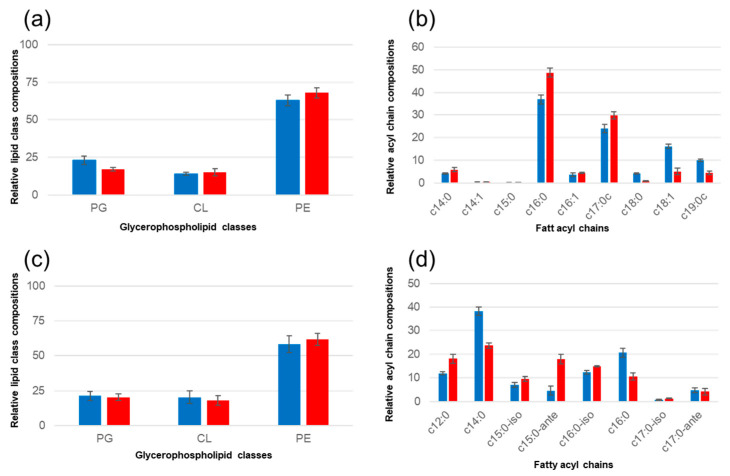
Analysis of protonated (blue) and perdeuterated (red) polar lipid mixtures extracted from *E. coli* (**a**,**b**) and *B. subtilis* (**c**,**d**). The graphics show the relative amount of the different lipid classes (left) and acyl chains (right) found in the natural polar lipid extracts, in mol %, as established by GC-MS. Error bars represent the variability between 3 replicates.

To approach a more physiologically relevant environment for membrane proteins, nanodiscs were assembled using natural polar lipid mixtures from Gram-negative (EcLip for *E. coli*) and Gram-positive (BsLip for *B. subtilis*) bacteria together with the csE3 belt protein. These nanodiscs possess a highly complex lipid composition (Figure 2), but their deuterated forms enable simplified SANS analysis, as the contribution of the lipid environment is matched out.

Both bacterial types feature a cytoplasmic membrane composed primarily of glycerophospholipids (GPLs) and a surrounding peptidoglycan layer. Gram-negative bacteria additionally have an outer membrane containing lipopolysaccharides. The cytoplasmic membrane of most bacterial species is dominated by three GPL classes: phosphatidylethanolamine (PE, the most abundant), phosphatidylglycerol (PG, anionic), and cardiolipin (CL), with relative proportions varying by species. Considerable variability also exists in fatty acyl chain composition across bacterial taxa.

In our analysis, consistent with prior reports [30,31], *B. subtilis* contained slightly less PE than *E. coli*, potentially affecting membrane surface charge. GC–FID analysis of the lipid extracts (Figure 2) showed that growth under deuterated conditions minimally affected the headgroup distribution but significantly altered fatty acyl chains, including chain length and unsaturation. Cells grown in D_2_O media produced GPLs with shorter and more saturated acyl chains compared to those grown in H_2_O.

In *E. coli*, the membrane lipids are dominated by saturated or cyclopropanated fatty acids of 14–18 carbons. Under deuterated growth, the relative abundances of stearic acid (18:0) and oleic acid (18:1) decreased markedly, while cyclopropanated fatty acids (19:0) also declined, accompanied by a modest increase in palmitic acid (16:0). These shifts, consistent with Corucci et al. [32], likely reflect adaptive mechanisms to maintain membrane fluidity under stronger D–D hydrogen bonding compared to H–H.

In contrast, *B. subtilis* membranes are rich in branched-chain fatty acids in both iso- and anteiso-configurations, typically saturated with a single methyl branch [33]. Branching at the penultimate carbon yields odd-chained iso-fatty acids, whereas branching at the antepenultimate carbon produces even-chained anteiso-fatty acids [34,35,36]. These branched chains confer greater membrane fluidity and a lower phase transition temperature, reducing lipid packing and membrane density relative to *E. coli* [31]. Under deuterated growth, lauric acid (12:0) increased, while myristic (14:0) and palmitic (16:0) decreased. Iso-branched fatty acids, notably iso-15:0 and iso-16:0, were elevated, alongside a pronounced increase in anteiso-15:0. These modifications also suggest adaptive responses to preserve fluidity under D_2_O conditions.

**Figure 3:**
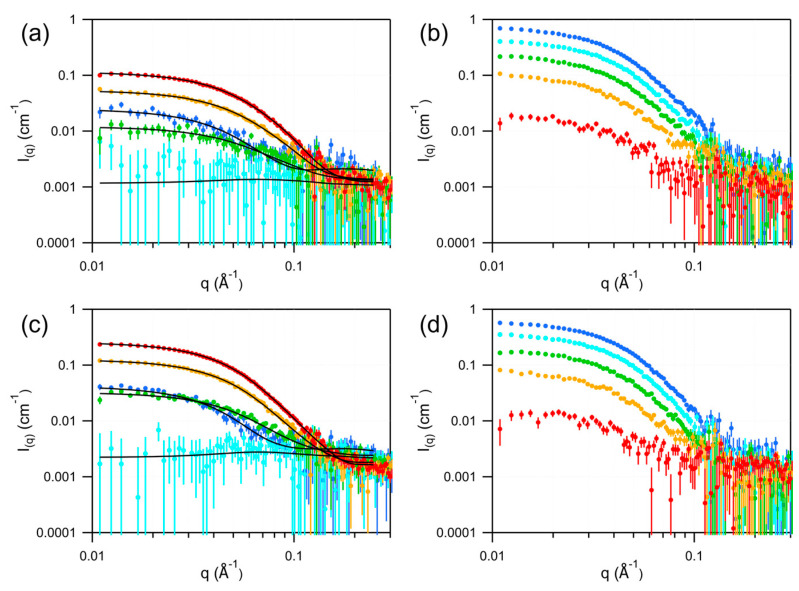
SANS analysis of the natural lipid mix nanodiscs. On the first line graph (**a**) concerns nanodiscs containing *E. coli* protonated lipids and graph (**b**), *E. coli* deuterated lipids. On the second line, graph (**c**) concerns nanodiscs containing *B. subtilis* protonated lipids, and graph (**d**), *B. subtilis* deuterated lipids. Colors represent the buffer D_2_O content: red: 80%; yellow: 60%; green: 40%; light blue: 20%; dark blue: 0%. The continuous black lines are the fits to the data obtained using SASView and correspond to the parameters listed in Table 1.

**Figure 4:**
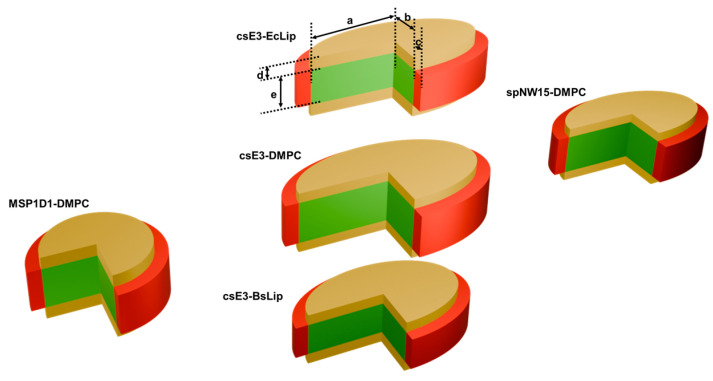
Schematic description of the nanodiscs characterized in this study. The red rings represent the belt proteins, the green discs, the lipid aliphatic chains and the yellow discs, the lipid headgroups. Being all depicted at the same scale, this representation enables a comparison between the different discs. Moreover, the legend shows distances reported in Table 1.

The protonated forms of the natural lipid nanodiscs were characterized by SANS using five contrast points. The resulting scattering curves were fitted under the same constraints applied to the hDMPC nanodiscs. Representative SANS curves and fits are shown in Figure 3a,c, with the corresponding numerical parameters listed in Table 1. As illustrated in Figure 4, nanodiscs assembled with natural lipids and the csE3 belt protein exhibit geometries comparable to those of the csE3–DMPC nanodiscs.

All nanodiscs are depicted to scale in Figure 4 using SANS-derived parameters, illustrating clear differences in size and shape: MSP1D1 nanodiscs are small and nearly circular, whereas csE3- and spNW15-defined nanodiscs are larger and elliptical, consistent with scaffold length controlling nanodisc size and prior DMPC-based SANS/SAXS studies [2,18]. Overall, the combined SAXS/SANS fitting strategy provides robust geometric characterization and suggests that csE3-defined nanodiscs offer a more stable environment for membrane protein studies compared to spNW15-based discs.

### 3.3. Deuterated Nanodiscs

**Figure 5:**
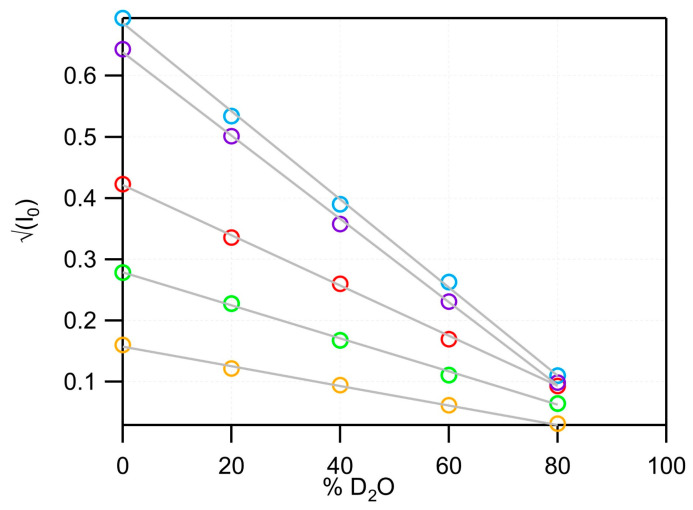
Plot of square root of I_(q=0)_ versus the percentage of D_2_O from the SANS curves of deuterated nanodiscs, enabling linear extrapolation of their match point: dMSP1D1-dDMPC (red, 102.8% D_2_O), dcsE3-dDMPC (orange, 98.3%), dspNW15-dDMPC (green, 103.3% D_2_O), dcsE3-dEcLip (blue, 95.3% D_2_O), and dcsE3-dBsLip (purple, 93.9% D_2_O). The differences in absolute values are due to different concentrations of each nanodisc, but the concentration is constant within each series.

To study membrane proteins reconstituted in nanodiscs by SANS, the most favorable contrast—maximizing the signal-to-noise ratio—is achieved when the protein of interest is protonated, while the nanodisc components are deuterated to match the SLD of a D_2_O-based buffer. Under these conditions, the nanodisc becomes effectively invisible, providing the highest contrast between the protein and its environment while minimizing incoherent scattering. Optimal contrast is obtained when the nanodisc belt protein is approximately 72% deuterated, and the lipids are fully deuterated. The five nanodisc variants described above were therefore assembled, purified, and measured by SANS at five contrast levels to experimentally determine their respective contrast match points. The scattering curves are shown in Figure 1b,e,i and Figure 3b,d. Match points were determined by plotting the square root of the coherent scattering intensity—calculated as the difference between a low-Q region (0.02–0.03 Å^−1^) and a high-Q region (0.2–0.3 Å^−1^)—against the percentage of D_2_O. As shown in Figure 5, the resulting match points were 102.8% D_2_O for dMSP1D1–dDMPC nanodiscs, 98.3% D_2_O for dcsE3–dDMPC, 103.3% D_2_O for dspNW15–dDMPC, 95.3% D_2_O for dcsE3–dEcLip, and 93.9% D_2_O for dcsE3–dBsLip nanodiscs. As expected, these results indicate that all nanodiscs can be effectively matched out at high D_2_O content, where the incoherent background is minimal, enabling optimal conditions for SANS measurements of protonated membrane proteins reconstituted within these lipid environments. Although these match points are not exactly 100% D_2_O, in classical conditions of biological samples, where the concentration is lower than 5 mg/mL, the remaining contribution of these nanodiscs will be smaller than the experimental noise.

## 4. Conclusions

This study provides an accurate characterization of nanodiscs assembled from three distinct belt proteins, along with insights into their relative stability, achieved through the simultaneous fitting of five SANS contrast points combined with three SAXS curves. Our results indicate that csE3-defined nanodiscs are more stable than those defined by spNW15. Although csE3 preparation involves enzymatic steps not required for spNW15, we recommend its use for membrane proteins that require 12 nm diameter discs. For smaller nanodiscs, circularized and negatively charged variants of MSP1D1 and MSP1ΔH5D1, as described by Barclay et al. [37], offer stable discs and will be incorporated into our library of deuterated belt proteins, expanding the available range down to ~6 nm diameter.

The geometric parameters obtained from SANS measurements of protonated nanodiscs provide a reference framework for future studies of nanodisc interactions. Furthermore, this work delivers a SANS-based structural description of nanodiscs containing natural lipid mixtures from Gram-positive bacteria, demonstrating that deuterated versions can be effectively matched out at high D_2_O content. This tunable environment enables the study of protonated membrane proteins under near-physiological conditions. Beyond bacterial lipid mixtures, deuterated yeast lipids [38] and chemically pure deuterated lipids [9] are also available, allowing selective inclusion of specific lipid species in either protonated or deuterated form to probe their effects on protein structure, dynamics, and interactions. The availability of these natural lipid mixtures allows the study of membrane proteins in an environment closer to their native context, although certain membrane properties, such as leaflet asymmetry, are still not fully recapitulated. While techniques such as crystallography and cryo-EM primarily reveal highly stable protein–lipid interactions, SANS expands the investigation to lower-affinity contexts. Although providing lower-resolution structural information, SANS complements high-resolution techniques and computational modeling to validate the solution behavior of membrane proteins.

## Figures and Tables

**Table 1:** Fit parameters and results. The values provided without error margins are not fitted. The values corresponding to several conditions are fitted but constrained to be the same in all conditions.

% D_2_O			Parameters (Å)					SLD (10^−6^ Å^−2^)				Χ^2^/Pts
			a-Radius	b/a-Radius Factor	c-Rim Thickness	d-Face Thickness	e-Length	Tails	Headgroups	Belt Protein	Solvent	
**SANS**	**MSP1D1-DMPC**	**0**	34.65 ± 1.18	0.99 ± 0.07	8.78 ± 0.08	8.44 ± 0.53	28.94 ± 0.08	−0.35 ± 0.01	1.89 ± 0.07	1.91	−0.55	0.89
		**20**							1.86 ± 0.24	2.15	0.83	0.66
		**40**							2.41 ± 0.14	2.38	2.22	0.90
		**60**							2.78 ± 0.19	2.61	3.60	1.36
		**80**							2.73 ± 0.30	2.85	4.99	1.03
	**csE3-DMPC**	**0**	65.13 ± 0.56	0.55 ± 0.01	9.79 ± 0.26	7.24 ± 0.19	31.89 ± 0.63	−0.39 ± 0.02	1.22 ± 0.12	1.91	−0.55	1.03
		**20**							1.97 ± 0.15	2.15	0.83	0.94
		**40**							1.94 ± 0.10	2.38	2.22	0.98
		**60**							2.21 ± 0.16	2.61	3.60	1.09
		**80**							2.34 ± 0.24	2.85	4.99	1.35
	**spNW15-DMPC**	**0**	54.13 ± 0.79	0.55 ± 0.02	8.89 ± 0.22	6.74 ± 0.68	29.54 ± 0.79	−0.37 ± 0.02	1.21 ± 0.22	1.92	−0.55	1.01
		**20**							1.69 ± 0.3	2.17	0.83	0.86
		**40**							1.82 ± 0.12	2.41	2.22	1.05
		**60**							1.88 ± 0.14	2.66	3.60	1.38
		**80**							1.50 ± 0.22	2.91	4.99	1.52
	**csE3-EcLip**	**0**	57.34 ± 0.90	0.47 ± 0.02	9.10 ± 0.83	7.99 ± 0.69	24.77 ± 1.51	−0.37 ± 0.01	2.81 ± 0.17	1.91	−0.55	0.95
		**20**							1.18 ± 0.42	2.15	0.83	0.64
		**40**							2.16 ± 0.17	2.38	2.22	0.70
		**60**							1.92 ± 0.19	2.61	3.60	0.70
		**80**							2.32 ± 0.26	2.85	4.99	1.01
	**csE3-BsLip**	**0**	57.84 ± 0.84	0.58 ± 0.02	9.50 ± 0.29	8.36 ± 0.59	26.07 ± 0.94	−0.39 ± 0.01	1.99 ± 0.05	1.91	−0.55	0.71
		**20**							1.52 ± 0.30	2.15	0.83	0.66
		**40**							2.02 ± 0.03	2.38	2.22	1.00
		**60**							2.19 ± 0.06	2.61	3.60	1.14
		**80**							2.99 ± 0.09	2.85	4.99	0.84
**SAXS**	**MSP1D1-DMPC**		39.26 ± 2.09	0.99 ± 0.00	8.99 ± 0.57	8.32 ± 0.41	30.86 ± 1.00	8.25 ± 0.08	12.27 ± 0.21	11.64 ± 0.05	9.40	1.27
	**csE3-DMPC**		68.52 ± 2.33	0.58 ± 0.07	8.56 ± 0.67					12.51 ± 0.29		1.82
	**spNW15-DMPC**		58.61 ± 4.69	0.55 ± 0.09	9.54 ± 0.77					11.73 ± 0.04		3.86

## Data Availability

SANS and SAXS data are available under DOI 10.5291/ILL-DATA.INTER-606.

## Abbreviations

The following abbreviations are used in this manuscript:

SANS	Small-angle neutron scattering
SAXS	Small-angle X-ray scattering
HPLC	High-performance liquid chromatography
DMPC	Di-myristoyl-phosphatidyl-choline
EcLip	*Escherichia coli* total polar lipids
BsLip	*Bacillus subtilis* total polar lipids
GC-MS	Gas chromatography coupled with mass spectrometry

## References

[1] White S.H. (2004). The Progress of Membrane Protein Structure Determination. Protein Sci..

[2] Denisov I.G., Grinkova Y.V., Lazarides A.A., Sligar S.G. (2004). Directed Self-Assembly of Monodisperse Phospholipid Bilayer Nanodiscs with Controlled Size. J. Am. Chem. Soc..

[3] Li Y., Kijac A.Z., Sligar S.G., Rienstra C.M. (2006). Structural Analysis of Nanoscale Self-Assembled Discoidal Lipid Bilayers by Solid-State NMR Spectroscopy. Biophys. J..

[4] Bayburt T.H., Sligar S.G. (2010). Membrane Protein Assembly into Nanodiscs. FEBS Lett..

[5] Shenkarev Z.O., Lyukmanova E.N., Paramonov A.S., Shingarova L.N., Chupin V.V., Kirpichnikov M.P., Blommers M.J.J., Arseniev A.S. (2010). Lipid−Protein Nanodiscs as Reference Medium in Detergent Screening for High-Resolution NMR Studies of Integral Membrane Proteins. J. Am. Chem. Soc..

[6] Zhang Y., Daday C., Gu R.-X., Cox C.D., Martinac B., De Groot B.L., Walz T. (2021). Visualization of the Mechanosensitive Ion Channel MscS under Membrane Tension. Nature.

[7] Golub M., Gätcke J., Subramanian S., Kölsch A., Darwish T., Howard J.K., Feoktystov A., Matsarskaia O., Martel A., Porcar L. (2022). “Invisible” Detergents Enable a Reliable Determination of Solution Structures of Native Photosystems by Small-Angle Neutron Scattering. J. Phys. Chem. B.

[8] Midtgaard S.R., Darwish T.A., Pedersen M.C., Huda P., Larsen A.H., Jensen G.V., Kynde S.A.R., Skar-Gislinge N., Nielsen A.J.Z., Olesen C. (2018). Invisible Detergents for Structure Determination of Membrane Proteins by Small-angle Neutron Scattering. FEBS J..

[9] Johansen N.T., Bonaccorsi M., Bengtsen T., Larsen A.H., Tidemand F.G., Pedersen M.C., Huda P., Berndtsson J., Darwish T., Yepuri N.R. (2022). Mg^2+^-Dependent Conformational Equilibria in CorA and an Integrated View on Transport Regulation. eLife.

[10] Gabel F. (2015). Small-Angle Neutron Scattering for Structural Biology of Protein–RNA Complexes. Methods in Enzymology.

[11] Breyton C., Gabel F., Lethier M., Flayhan A., Durand G., Jault J.-M., Juillan-Binard C., Imbert L., Moulin M., Ravaud S. (2013). Small Angle Neutron Scattering for the Study of Solubilised Membrane Proteins. Eur. Phys. J. E.

[12] Sugiyama M., Yagi H., Ishii K., Porcar L., Martel A., Oyama K., Noda M., Yunoki Y., Murakami R., Inoue R. (2016). Structural Characterization of the Circadian Clock Protein Complex Composed of KaiB and KaiC by Inverse Contrast-Matching Small-Angle Neutron Scattering. Sci. Rep..

[13] Lapinaite A., Carlomagno T., Gabel F., Arluison V., Wien F. (2020). Small-Angle Neutron Scattering of RNA–Protein Complexes. RNA Spectroscopy.

[14] Combet S., Bonneté F., Finet S., Pozza A., Saade C., Martel A., Koutsioubas A., Lacapère J.-J. (2023). Effect of Amphiphilic Environment on the Solution Structure of Mouse TSPO Translocator Protein. Biochimie.

[15] Maric S., Skar-Gislinge N., Midtgaard S., Thygesen M.B., Schiller J., Frielinghaus H., Moulin M., Haertlein M., Forsyth V.T., Pomorski T.G. (2014). Stealth Carriers for Low-Resolution Structure Determination of Membrane Proteins in Solution. Acta Crystallogr. D Biol. Crystallogr..

[16] Josts I., Nitsche J., Maric S., Mertens H.D., Moulin M., Haertlein M., Prevost S., Svergun D.I., Busch S., Forsyth V.T. (2018). Conformational States of ABC Transporter MsbA in a Lipid Environment Investigated by Small-Angle Scattering Using Stealth Carrier Nanodiscs. Structure.

[17] Johansen N.T., Tidemand F.G., Nguyen T.T.T.N., Rand K.D., Pedersen M.C., Arleth L. (2019). Circularized and Solubility-enhanced MSP s Facilitate Simple and High-yield Production of Stable Nanodiscs for Studies of Membrane Proteins in Solution. FEBS J..

[18] Zhang S., Ren Q., Novick S.J., Strutzenberg T.S., Griffin P.R., Bao H. (2021). One-Step Construction of Circularized Nanodiscs Using SpyCatcher-SpyTag. Nat. Commun..

[19] Cai M., Huang Y., Craigie R., Clore G.M. (2019). A Simple Protocol for Expression of Isotope-Labeled Proteins in Escherichia Coli Grown in Shaker Flasks at High Cell Density. J. Biomol. NMR.

[20] Bligh E.G., Dyer W.J. (1959). A rapid method of total lipid extraction and purification. Can. J. Biochem. Physiol..

[21] Folch J., Lees M., Sloane Stanley G.H. (1957). A Simple Method for the Isolation and Purification of Total Lipides from Animal Tissues. J. Biol. Chem..

[22] Nakanishi H., Hayashida K., Nishizawa T., Oshima A., Abe K. (2022). Cryo-EM of the ATP11C Flippase Reconstituted in Nanodiscs Shows a Distended Phospholipid Bilayer Inner Membrane around Transmembrane Helix 2. J. Biol. Chem..

[23] Tully M.D., Kieffer J., Brennich M.E., Cohen Aberdam R., Florial J.B., Hutin S., Oscarsson M., Beteva A., Popov A., Moussaoui D. (2023). BioSAXS at European Synchrotron Radiation Facility—Extremely Brilliant Source: BM29 with an Upgraded Source, Detector, Robot, Sample Environment, Data Collection and Analysis Software. J. Synchrotron Rad..

[24] Kieffer J., Brennich M., Florial J.-B., Oscarsson M., De Maria Antolinos A., Tully M., Pernot P. (2022). New Data Analysis for BioSAXS at the ESRF. J. Synchrotron Rad..

[25] Larsen A.H., Pedersen M.C. (2021). Experimental Noise in Small-Angle Scattering Can Be Assessed Using the Bayesian Indirect Fourier Transformation. J. Appl. Crystallogr..

[26] Nakano M., Fukuda M., Kudo T., Miyazaki M., Wada Y., Matsuzaki N., Endo H., Handa T. (2009). Static and Dynamic Properties of Phospholipid Bilayer Nanodiscs. J. Am. Chem. Soc..

[27] Kumari P., Faraone A., Kelley E.G., Benedetto A. (2021). Stiffening Effect of the [Bmim][Cl] Ionic Liquid on the Bending Dynamics of DMPC Lipid Vesicles. J. Phys. Chem. B.

[28] Heller W.T. (2022). Small-Angle Neutron Scattering for Studying Lipid Bilayer Membranes. Biomolecules.

[29] Harvey R.D., Bello G., Kikhney A.G., Torres J., Surya W., Wölk C., Shen C. (2023). Absolute Scattering Length Density Profile of Liposome Bilayers Obtained by SAXS Combined with GIXOS: A Tool to Determine Model Biomembrane Structure. J. Appl. Crystallogr..

[30] Gidden J., Denson J., Liyanage R., Ivey D.M., Lay J.O. (2009). Lipid Compositions in Escherichia Coli and Bacillus Subtilis during Growth as Determined by MALDI-TOF and TOF/TOF Mass Spectrometry. Int. J. Mass. Spectrom..

[31] Barák I., Muchová K. (2013). The Role of Lipid Domains in Bacterial Cell Processes. Int. J. Mol. Sci..

[32] Corucci G., Vadukul D.M., Paracini N., Laux V., Batchu K.C., Aprile F.A., Pastore A. (2025). Membrane Charge Drives the Aggregation of TDP-43 Pathological Fragments. J. Am. Chem. Soc..

[33] DiomandÃ© S.E., Nguyen-The C., GuinebretiÃ¨re M.-H., Broussolle V., Brillard J. (2015). Role of Fatty Acids in Bacillus Environmental Adaptation. Front. Microbiol..

[34] Kaneda T. (1967). Fatty Acids in the Genus *Bacillus* I. Iso- and Anteiso-Fatty Acids as Characteristic Constituents of Lipids in 10 Species. J. Bacteriol..

[35] Kates M. (1964). Bacterial Lipids. Adv. Lipid Res..

[36] Macfarlane M.G. (1961). Cardiolipin and Other Phospholipids in Ox Liver. Biochem. J..

[37] Barclay A., Tidemand Johansen N., Tidemand F.G., Arleth L., Pedersen M.C. (2022). Global Fitting of Multiple Data Frames from SEC–SAXS to Investigate the Structure of next-Generation Nanodiscs. Acta Crystallogr. D Struct. Biol..

[38] De Ghellinck A., Schaller H., Laux V., Haertlein M., Sferrazza M., Maréchal E., Wacklin H., Jouhet J., Fragneto G. (2014). Production and Analysis of Perdeuterated Lipids from Pichia Pastoris Cells. PLoS ONE.

